# Cardiac Amyloidosis: A Narrative Review of Diagnostic Advances and Emerging Therapies

**DOI:** 10.3390/biomedicines13051230

**Published:** 2025-05-19

**Authors:** Dana Emilia Movila, Alexandru Catalin Motofelea, Dragos Cozma, Oana Albai, Alexandra Christa Sima, Minodora Andor, Tudor Ciocarlie, Simona Ruxanda Dragan

**Affiliations:** 1 University Clinic of Internal Medicine and Ambulatory Care, Prevention and Cardiovascular Recovery, Department VI—Cardiology, “Victor Babes” University of Medicine and Pharmacy, 300041 Timisoara, Romania; man.dana@umft.ro (D.E.M.); simona.dragan@umft.ro (S.R.D.); 2 Research Centre of Timisoara Institute of Cardiovascular Diseases, “Victor Babes” University of Medicine and Pharmacy, 300041 Timisoara, Romania; dragos.cozma@umft.ro; 3 Institute of Cardiovascular Diseases Timisoara, 300310 Timisoara, Romania; 4 Centre for Molecular Research in Nephrology and Vascular Disease/MOL-NEPHRO-VASC, “Victor Babes” University of Medicine and Pharmacy, 300041 Timisoara, Romania; albai.oana@umft.ro (O.A.); sima.alexandra@umft.ro (A.C.S.); 5 Department of Second Internal Medicine—Diabetes, Nutrition, Metabolic Diseases, and Systemic Rheumatology, “Victor Babes” University of Medicine and Pharmacy, 300041 Timisoara, Romania; 6 Department of Diabetes, Nutrition and Metabolic Diseases Clinic, “Pius Brînzeu” Emergency Clinical County University Hospital, 300723 Timisoara, Romania; 7 Discipline of Medical Semiotics II, Department V—Internal Medicine-1, “Victor Babes” University of Medicine and Pharmacy, 300041 Timisoara, Romania; andor.minodora@umft.ro; 8 Department VII Internal Medicine II, Discipline of Cardiology, University of Medicine and Pharmacy “Victor Babes”, 300041 Timisoara, Romania; ciocarlie.tudor@umft.ro

**Keywords:** amyloidosis, cardiac amyloidosis, diagnosis, TTR, transthyretin

## Abstract

**Background/Objectives**: Cardiac amyloidosis (CA) is an underdiagnosed and potentially life-threatening infiltrative cardiomyopathy characterized by the extracellular deposition of misfolded amyloid fibrils in cardiac tissue. It is most commonly associated with light-chain (AL) amyloidosis and transthyretin (ATTR) amyloidosis, either hereditary or wild-type. The disease often presents with non-specific symptoms, leading to delayed diagnosis and treatment. This study aims to provide a comprehensive overview of the pathophysiology, diagnostic strategies, and current therapeutic approaches for cardiac amyloidosis, with a focus on improving early detection and clinical outcomes. **Methods**: A narrative review was conducted using databases such as PubMed and Scopus, covering the period from September 2016 to March 2025. Keywords such as “cardiac amyloidosis”, “cardiac amyloidosis from transthyretin”, “cardiomyopathy”, “transthyretin”, “immunoglobulin light-chain amyloidosis”, and “familial amyloidosis” were used. Relevant clinical trials and guideline-based management recommendations were also included. **Results**: This review highlights that non-invasive imaging modalities and serum biomarker analyses are key to reducing diagnostic delays. New therapeutic developments, including gene-editing technologies and RNA-based therapies, show promise in early trials. Multidisciplinary management and increased awareness are crucial for timely diagnosis and treatment optimization. **Conclusions**: The early recognition of cardiac amyloidosis remains a major clinical challenge. Advances in non-invasive diagnostics and emerging disease-modifying therapies are transforming the prognosis of affected patients. Continued research and heightened clinical suspicion are essential to improve outcomes in this complex and heterogeneous disease.

## 1. Introduction

Amyloidosis is a disorder characterized by the extracellular accumulation of fibrils composed of low-molecular-weight subunits derived from various serum proteins. This condition can affect multiple organs, with cardiac, renal, hepatic, and autonomic nervous system involvements being the primary contributors to morbidity and mortality. Several factors increase the risk of developing amyloidosis, including advanced age, male sex, African ancestry, chronic or infectious diseases, and a family history of the condition, as certain types of amyloidosis are hereditary [1]. Moreover, the transthyretin (TTR) Ile122 variant—strongly associated with cardiac amyloidosis (CA) in individuals of African descent—has an allele frequency of 66/3376 (2.0%) among African-Americans across the U.S., suggesting it is a common, often unrecognized contributor to cardiac disease in this population [2].

CA is characterized by the extracellular buildup of misfolded proteins within the heart, marked by a unique histological feature: green birefringence under cross-polarized light following Congo red staining. CA involves the extracellular accumulation of fibrillar and insoluble protein aggregates within the myocardium, leading to cardiac dysfunction [3]. Although over 30 types of amyloidogenic proteins have been identified [4], five of them affect the heart, including immunoglobulin heavy and light chain (AL), transthyretin (TTR), amyloid A, and apolipoprotein A1. Among these, the AL and ATTR types (wild-type [ATTRwt] and hereditary/variant [ATTRv]) account for 95% of all CA cases [5]. The pathogenesis of CA starts with the misfolding of precursor proteins, which form insoluble fibrils that deposit throughout the myocardial interstitium and perivascular spaces, ultimately disrupting the normal tissue architecture and compliance [6,7]. As these deposits accumulate, the heart develops a restrictive physiology characterized by impaired ventricular filling and, over time, systolic dysfunction [8]. Despite its clinical significance, CA is often underdiagnosed: the systematic screening of older adults with heart failure and preserved ejection fraction (HFpEF) has revealed CA in up to 16% of these patients, underscoring a significant diagnostic gap in this population [9].

The infiltrative process in the heart results in progressive myocardial dysfunction, often accompanied by the impairment of the cardiac conduction system. Amyloid cardiomyopathy is increasingly recognized as a significant yet frequently underdiagnosed cause of heart failure and cardiac arrhythmias, particularly in older adults. In parallel with mechanical impairment, amyloid fibrils infiltrate the cardiac conduction system—including the sinoatrial and atrioventricular nodes and the His–Purkinje network—creating areas of conduction block and promoting reentrant circuits [9]. As a result, atrial fibrillation (AF) arises in up to 73% of patients with transthyretin amyloidosis, especially those of an advanced age, a higher disease stage, and enlarged left atrial volumes [10]. The combination of a poor rate control tolerance and a markedly elevated stroke risk makes AF management challenging; current evidence supports early rhythm-control strategies and indicates that novel oral anticoagulants provide a thromboembolic protection comparable to warfarin with fewer major bleedings, while timely AF ablation may reduce both mortality and heart-failure hospitalizations [10,11]. Conduction blocks and ventricular tachyarrhythmias also occur frequently, often necessitating permanent pacing for symptomatic bradyarrhythmias or advanced atrioventricular blocks [12]. Although implantable cardioverter-defibrillators (ICDs) are used for sudden cardiac death (SCD) prevention, their impact on the overall survival in CA remains uncertain, as a high mortality persists despite device therapy [13].

Although traditionally regarded as a rare condition, emerging evidence indicates that cardiac amyloidosis is often overlooked as a contributing factor to common cardiac diseases and syndromes. Recent advancements in cardiac imaging, diagnostic techniques, and therapeutic approaches have significantly enhanced the identification and management of this condition [14].

Advancements in cardiac imaging and increased physician awareness have significantly improved the diagnosis of cardiac amyloidosis over the past decade. Cardiac involvement in amyloidosis is associated with a poor prognosis, making early detection essential. A timely diagnosis is critical for implementing appropriate treatment strategies that can potentially alter the disease’s natural course and improve patient outcomes [15]. This review highlights that non-invasive imaging modalities and serum biomarker analyses are key to reducing diagnostic delays. New therapeutic developments, including gene-editing technologies and RNA-based therapies, show promise in early trials. Multidisciplinary management and increased awareness are crucial for timely diagnosis and treatment optimization.

## 2. Materials and Methods

This work was designed as a narrative review, with its primary focus being cardiac amyloidosis. A comprehensive literature search was independently performed by two researchers using the PubMed and Scopus databases from September 2016 to March 2025. The search strategy included a range of relevant keyword combinations, such as “cardiac amyloidosis”, “cardiac amyloidosis from transthyretin”, “cardiomyopathy”, “transthyretin”, “immunoglobulin light-chain amyloidosis”, and “familial amyloidosis”.

Review articles and meta-analyses were included. Articles were excluded based on the following criteria: non-English language publications, case reports, editorials, or studies lacking relevance to the review topic. After applying the exclusion criteria, a total of 37 studies were included in the final review.

## 3. Clinical and Multimodal Imaging Features of Cardiac Amyloidosis

The diagnostic assessment of a patient with suspected cardiac amyloidosis begins with a comprehensive clinical evaluation—a detailed history and physical examination—to identify and assess the key cardiac and extracardiac manifestations of **AL and ATTR** amyloidosis, as summarized in Table 1 [16].

The diagnosis of cardiac amyloidosis requires a high level of clinical suspicion but is often delayed due to limited disease awareness and the diverse range of presenting symptoms. Many patients remain asymptomatic until the disease has significantly progressed, and even at that stage, symptoms are often non-specific, further complicating early detection [17].

The key cardiac findings are summarized in Table 2. One of the hallmark electrocardiographic features raising the suspicion of cardiac amyloidosis is disproportionately low QRS voltages relative to the left ventricular (LV) wall thickness. Echocardiography serves as the initial imaging modality for suspected cardiac amyloidosis and can reveal various abnormalities, including LV hypertrophy with or without right ventricular (RV) free wall hypertrophy, bi-atrial dilation, myocardial granular sparkling on non-harmonic imaging, thickened mitral and tricuspid valve leaflets, atrial septal thickening, low-flow/low-gradient aortic stenosis, diastolic dysfunction of varying severity, and abnormal left ventricular global longitudinal strain, typically exhibiting an apical sparing pattern [17,18].

Further tissue characterization using cardiovascular magnetic resonance (CMR) imaging may reveal a diffuse subendocardial or transmural late gadolinium enhancement, along with an increased extracellular volume as assessed by T1 mapping, providing additional diagnostic insight [19].

## 4. Diagnostic Criteria for Cardiac Amyloidosis

Although over thirty proteins are known to aggregate as amyloid in vivo, only nine of these amyloidogenic proteins accumulate in the myocardium and lead to clinically significant cardiac disease.

However, certain forms of cardiac amyloidosis, such as AApoAI, AApoAII, AApoAIV, Aβ2M, AFib, and AGel, are extremely rare. Additionally, cardiac amyloidosis secondary to chronic inflammatory or infectious diseases (AA) has become much less common [20,21,22]. Currently, over 98% of diagnosed cases of cardiac amyloidosis are attributed to fibrils composed of either monoclonal immunoglobulin light chains (AL) or transthyretin (ATTR), which can occur in hereditary (ATTRv) or acquired (ATTRwt) forms [22]. Table 1 outlines the key characteristics of each type of cardiac amyloidosis.

Cardiac amyloidosis is confirmed by the presence of amyloid fibrils within cardiac tissue. Diagnostic criteria can be categorized into invasive and non-invasive methods. The invasive criteria, which require a histological confirmation of amyloid through a biopsy, apply to all CA subtypes. In contrast, the non-invasive criteria—characterized by typical echo/CMR findings combined with Grade 2–3 uptake on 99mTc-PYP/DPD/HMDP scintigraphy and the exclusion of a monoclonal protein—are validated exclusively for ATTR amyloidosis [21].

### 4.1. Non-Invasive Diagnostic Criteria

Cardiac ATTR amyloidosis can be diagnosed without histological confirmation if typical echocardiographic or cardiac magnetic resonance (CMR) findings are present, and scintigraphy with 99mTc-Pyrophosphate (PYP), 99mTc-DPD, or 99mTc-HMDP shows a Grade 2 or 3 myocardial radiotracer uptake. Additionally, clonal dyscrasia must be ruled out through the following tests: serum-free light chain (FLC) assay, serum protein electrophoresis with immunofixation (SPIE), and urine protein electrophoresis with immunofixation (UPIE). The combined use of SPIE, UPIE, and serum FLC quantification achieves a 99% sensitivity for identifying abnormal pro-amyloidotic precursors in AL amyloidosis. Importantly, serum and urine electrophoresis should always include immunofixation to enhance sensitivity for detecting monoclonal proteins [23,24]. The initial diagnostic steps involve nuclear scintigraphy using 99mTc-PYP, 99mTc-DPD, or 99mTc-HMDP to assess myocardial uptake, alongside a monoclonal protein assessment through serum protein electrophoresis with immunofixation (SPIE), urine protein electrophoresis with immunofixation (UPIE), and the quantification of serum-free light chains (FLCs) [25,26]. The interpretation of results involves identifying AL amyloidosis in cases with positive monoclonal proteins, necessitating a hematologic evaluation and confirmatory biopsy, while the absence of monoclonal proteins with Grade 2/3 uptake on scintigraphy suggests ATTR amyloidosis, requiring genetic testing to differentiate between wild-type (ATTRwt) and variant (ATTRv) forms. If results remain inconclusive, an endomyocardial or extracardiac biopsy should be performed for a definitive diagnosis and amyloid typing. This structured diagnostic approach ensures the accurate identification of cardiac amyloidosis subtypes, facilitating the timely initiation of targeted treatment strategies [27], based on the results of scintigraphy and monoclonal protein assessments.

### 4.2. Invasive Diagnostic Criteria

**Figure 1:**
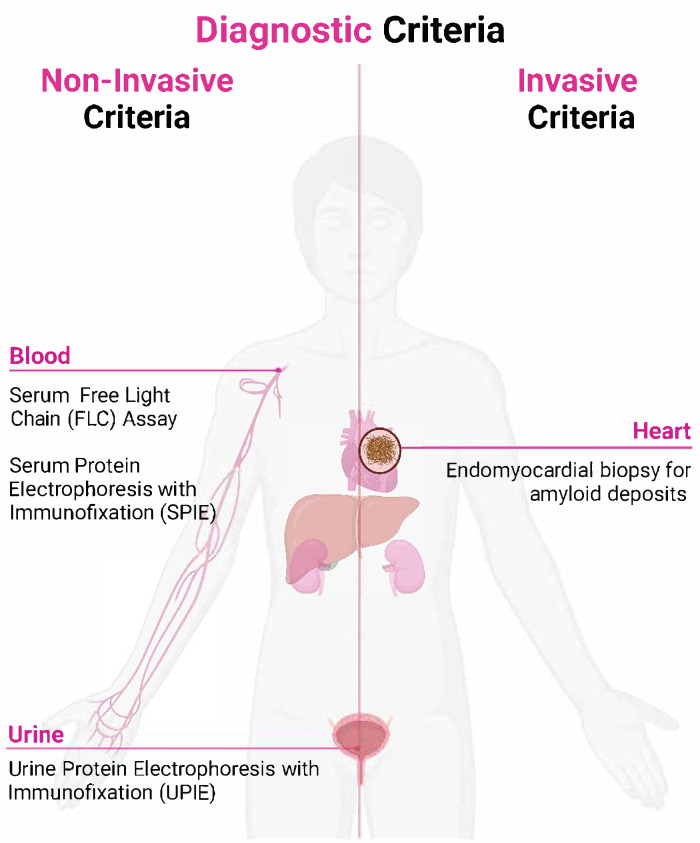
Diagnostic criteria for cardiac amyloidosis.

Cardiac amyloidosis is confirmed through an endomyocardial biopsy that reveals amyloid deposits via Congo red staining, irrespective of the extent of the left ventricular (LV) wall thickness (Figure 1). A significant number of patients with CA require tissue sampling for diagnosis, and histological typing can provide prognostic information and guide treatment [28]. Treatments are available for many types of amyloid but are specific to each type; thus, the confirmation and typing of amyloid are crucial before starting therapy [29]. Patients with AL amyloidosis are particularly susceptible to the cardiovascular toxicity associated with AL-directed chemotherapy (daratumumab-CyBorD) [30].

Upon the detection of amyloid deposits, precise fibril typing is essential to guide therapy: a laser-capture microdissection coupled with mass spectrometry-based proteomics is now considered the gold standard for amyloid typing, offering unparalleled sensitivity and specificity [31]. While immunohistochemistry and immunoelectron microscopy remain valuable, more widely available methods for routine classification in specialized centers is common [23]. Once amyloid is detected, the fibril protein must be classified. While mass spectrometry remains the gold standard for amyloid typing, immunohistochemistry and immunoelectron microscopy are commonly used in specialized centers for routine identification [23].

A diagnosis can also be established if amyloid deposits are identified in an extracardiac biopsy, provided there are characteristic features of cardiac amyloidosis observed on echocardiography—without another explanation for an increased LV wall thickness or distinctive findings in cardiac magnetic resonance (CMR) imaging [24,32,33].

A recent multicenter study proposed an echocardiographic scoring system to aid in diagnosing AL or ATTR amyloidosis in cases of increased LV wall thickness. Although not yet externally validated, a score of ≥ 8 points, combined with an LV wall thickness ≥ 12 mm and amyloid deposits confirmed by an extracardiac biopsy, may also be considered diagnostic for cardiac amyloidosis [32,33,34].

### 4.3. Challenges in Interpretation

Low-level monoclonal proteins or mild abnormalities in the kappa–lambda FLC ratio can occur in patients with chronic kidney disease (CKD) or monoclonal gammopathy of undetermined significance (MGUS). In CKD, a reduced glomerular filtration rate (GFR) leads to the decreased renal clearance of polyclonal FLCs, causing serum levels to rise. The FLC ratio varies based on the assay used. For the Freelite Assay (Binding Site), the FLC ratio increases with a declining GFR. A range of 0.37–3.1 is considered normal for patients with CKD. For the N Latex Assay (Siemens), the FLC ratio decreases as the GFR declines, but no reference range has been proposed for CKD. For patients with moderate CKD (eGFR < 45 mL/min/1.73 m^2^ using the CKD-EPI formula), a FLC ratio up to 2.0 (or 3.1 in dialysis) can typically be regarded as normal in the context of normal SPIE/UPIE results [24,25].

If FLC findings are unclear or abnormal, a consultation with a hematologist is recommended for further evaluation. In the absence of a detectable monoclonal protein and an abnormal serum FLC ratio, the specificity of Grade 2 or 3 bone scintigraphy for diagnosing cardiac ATTR amyloidosis in suspected cases is estimated to be nearly 100%. However, it is crucial to perform scintigraphy with single-photon emission computed tomography (SPECT) to ensure that the observed uptake corresponds to the myocardium and not the cardiac chambers [35]. It is important to note that rare conditions may also cause a positive cardiac uptake on scintigraphy. These alternative causes should always be considered when interpreting the results to avoid misdiagnosis [36]. Once cardiac ATTR amyloidosis is confirmed, genetic counseling and testing should be conducted to identify TTR mutations, enabling a differentiation between wild-type ATTR (ATTRwt) and hereditary ATTR (ATTRv). Genetic testing is recommended even in elderly patients, as a notable proportion may carry TTR mutations [37,38,39].

The diagnostic process for cardiac amyloidosis involves two key phases:

- Suspicion phase: identifying clinical and imaging features that raise suspicion for cardiac amyloidosis.
- Definitive diagnosis phase: confirming the presence of amyloid deposits and accurately typing the amyloid fibrils to guide targeted treatments [40].

**Figure 2:**
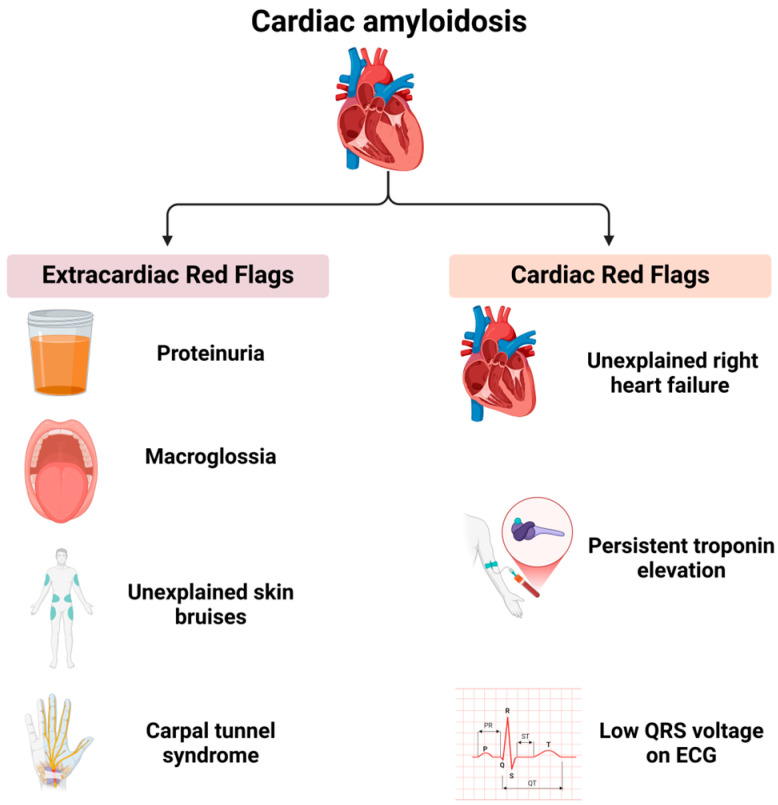
Red flags in cardiac amyloidosis.

Cardiac amyloidosis is frequently associated with extracardiac manifestations that, in conjunction with characteristic cardiac imaging findings, can significantly raise clinical suspicion. These manifestations, referred to as “red flags”, serve as critical indicators for the early recognition of the disease (Figure 2).

Extracardiac red flags include proteinuria (even if mild), macroglossia (enlarged tongue), unexplained skin bruising, and carpal tunnel syndrome. Cardiac red flags comprise heart failure with disproportionately elevated N-terminal pro-B-type natriuretic peptide (NT-proBNP) levels relative to echocardiographic findings, unexplained right heart failure despite normal ventricular and valvular function, idiopathic pericardial effusion, persistent troponin elevation, a low QRS voltage on an electrocardiogram (ECG) disproportionate to the left ventricular (LV) wall thickness, and an early-onset conduction system disease. The recognition of these extracardiac and cardiac red flags should prompt a further evaluation for cardiac amyloidosis, particularly when imaging findings are suggestive of the condition [41,42,43,44]. Beyond specific red flags, certain clinical scenarios warrant a heightened suspicion for cardiac amyloidosis. Cardiac disease occurring in the context of a systemic condition, such as plasma cell dyscrasia, nephrotic syndrome, peripheral neuropathy, or chronic systemic inflammatory conditions, should prompt a thorough diagnostic workup for amyloidosis when accompanied by compatible cardiac imaging findings. An increased left ventricular wall thickness in a non-dilated left ventricle is a hallmark feature of cardiac amyloidosis and necessitates further investigation, particularly in elderly patients presenting with heart failure with preserved ejection fraction (HFpEF), hypertrophic cardiomyopathy, or severe aortic stenosis. Patients undergoing a transcatheter aortic valve replacement (TAVR) should be evaluated for cardiac amyloidosis, given that transthyretin (ATTR) amyloidosis has been identified in 7–19% of such cases [44]. Non-invasive diagnostic methods facilitate early identification, and individuals with an increased wall thickness presenting with heart failure, aortic stenosis, or red flag symptoms should be assessed for cardiac amyloidosis, particularly those over the age of 65 [45].

About 5% of patients with a history of bilateral carpal tunnel syndrome are also later found to have cardiac amyloidosis [46].

The early differentiation of AL versus ATTR amyloidosis is essential. Since most cases of cardiac amyloidosis are attributed to AL or ATTR subtypes, the diagnostic strategy primarily focuses on distinguishing between these forms. Begin with serum and urine immunofixation plus free light-chain quantification to detect a monoclonal protein, and perform bone scintigraphy (99mTc-PYP/DPD/HMDP) for myocardial uptake. A positive monoclonal protein directs the patient to a hematology and biopsy confirmation for AL amyloidosis. In contrast, an isolated Grade 2–3 tracer uptake without a monoclonal protein confirms ATTR amyloidosis and prompts genetic testing (ATTRv vs. ATTRwt). If either pathway remains equivocal, proceed to an endomyocardial or extracardiac biopsy with amyloid typing. Table 3 summarizes these four diagnostic scenarios.

## 5. Discussion

Cardiac amyloidosis (CA) is a complex and progressive infiltrative cardiomyopathy caused by the extracellular deposition of amyloid fibrils within myocardial tissue, leading to restrictive physiology and progressive heart failure. The early recognition and differentiation of subtypes are critical for guiding treatment and improving outcomes in this frequently underdiagnosed condition.

The main subtypes of cardiac amyloidosis include immunoglobulin light-chain (AL) amyloidosis and transthyretin (ATTR) amyloidosis. AL amyloidosis arises from a clonal plasma cell disorder producing misfolded light chains that deposit systemically, often associated with multiple myeloma. In contrast, ATTR amyloidosis results from misfolded transthyretin proteins, which can be inherited (variant, ATTRv) or acquired (wild-type, ATTRwt) forms [1,14]. While AL amyloidosis progresses rapidly and is potentially fatal without prompt therapy, ATTR amyloidosis often has a more indolent course, particularly the wild-type form.

Clinically, CA may present with non-specific symptoms, such as exertional dyspnea, fatigue, edema, and signs of heart failure with preserved ejection fraction (HFpEF), contributing to diagnostic delays [47,48]. Arrhythmias, particularly atrial fibrillation and conduction abnormalities, are also common. Extracardiac manifestations—including neuropathy, carpal tunnel syndrome, and nephrotic-range proteinuria—may offer important diagnostic clues.

A systematic review and meta-analysis by Antonopoulos et al. (2022) [49] confirmed that ATTR-CA is the most common form of cardiac amyloidosis, although AL-CA may account for up to 18% of cases. The average age at diagnosis is typically between 74 and 90 years, with a clear male predominance (50–100%). ATTR-CA is more prevalent than previously believed, particularly among patients with HFpEF, severe aortic stenosis (AS), or hypertrophic cardiomyopathy [49].

The diagnostic approach to CA is multimodal. Echocardiography (ECHO) is generally the first-line tool, but findings such as an increased wall thickness, elevated left ventricular mass index (LVMI), and diastolic dysfunction are often non-specific and may overlap with hypertensive heart disease or hypertrophic cardiomyopathy [14,50]. Jaiswal et al. (2022) [51] demonstrated that subtle echocardiographic differences in wall thickness and diastolic parameters may help screen AS patients for CA. Still, optimal thresholds for these markers require further validation [51].

Advanced cardiac imaging has substantially improved diagnostic accuracy. Cardiac magnetic resonance imaging (CMR) with late gadolinium enhancement enables the visualization of amyloid infiltration and tissue characterization. Tissue mapping techniques—such as T1 mapping and extracellular volume (ECV) quantification—have prognostic value. Studies found that elevated native T1 times, a higher ECV, and lower myocardial-to-skeletal T2 ratios correlate with worse outcomes, reinforcing the importance of CMR in risk stratification [52].

Positron emission tomography (PET), particularly using tracers like florbetapir, is a promising modality in CA diagnosis. A systematic review by Kim et al. (2020) [53] highlighted the utility of PET, especially when paired with a semi-quantitative analysis, in differentiating between AL and ATTR amyloidosis. These results support the use of PET alongside CMR and bone scintigraphy to enhance diagnostic precision [53].

An invasive tissue biopsy remains the gold standard for diagnosis, with Congo red staining and immunohistochemical subtyping performed on samples from the abdominal fat pad, rectal mucosa, or endomyocardial tissue. While a fat pad biopsy is less invasive, it may have a reduced sensitivity in ATTR cases, necessitating a myocardial biopsy in selected patients [54,55]. Fine-needle fat aspiration is integrated into CA diagnosis, reliably documenting amyloid deposits and indicating cardiac involvement when combined with imaging [56]. The sensitivity is 85.1% and specificity is 97.1% under optimal conditions [40]. The abdominal fat pad excisional biopsy (FPEB) and fat fine-needle biopsy identify and classify CA, offering simplicity, low costs, and minimal complications [56]. In AL-CA patients, the abdominal fat fine-needle aspiration shows a 100% specificity and an 84% sensitivity, though a lower sensitivity for ATTR (45% for ATTRv and 15% for ATTRwt) [56]. Fine-needle aspiration provides better material for amyloid typing than FPEB. FPEB’s sensitivity for AL amyloidosis varies from 50% (<700 mm^3^) to 100% (>700 mm^3^) [56,57]. The initial surgical sample should be 1400 mm3 to allow division for immunofluorescence, mass spectrometry, and electron microscopy studies.

The importance of subtype differentiation is underscored by Xin et al. (2019) [58], who demonstrated the distinct prognostic implications of AL vs. ATTR cardiac amyloidosis. AL amyloidosis requires urgent chemotherapy targeting the plasma cell clone, while ATTR amyloidosis may be managed with transthyretin-stabilizing agents. Tafamidis and patisiran, for example, have demonstrated survival benefits in ATTR-CA, particularly in early stages of the disease [1,49]. Diflunisal and inotersen are also being considered as therapeutic options in clinical evaluations; however, their substantial out-of-pocket and drug acquisition costs continue to be significant barriers to their widespread adoption.

Heart failure management in CA is complex. Diuretics are used cautiously for volume overload, but beta-blockers and ACE inhibitors may not be well tolerated due to low-output states. Anticoagulation is indicated in patients with atrial fibrillation or left atrial dysfunction to prevent thromboembolic events [59]. Patients with bradyarrhythmias or a conduction disease may require pacemakers or defibrillators. For those with obstructive symptoms, septal alcohol ablation has been explored, although the restrictive physiology complicates procedural success [60]. Electrophysiological studies and catheter ablation may provide symptomatic relief in selected cases [61,62].

In patients with severe AS undergoing a transcatheter aortic valve replacement (TAVR), concomitant ATTR-CA is found in up to 13.3% of cases [63]. These individuals face higher mortality and cardiovascular hospitalization rates compared to AS patients without amyloidosis. Recent studies noted that the left ventricular wall thickness (LVWT) independently predicts mortality in AS-CA patients, regardless of ejection fraction or surgical interventions. The authors advocate for randomized controlled trials to evaluate the benefit of combining an amyloid-specific therapy with standard valve treatments [64,65].

Despite these advancements, CA remains underdiagnosed. Heightened clinical suspicion in high-risk populations, improved access to advanced imaging, and multidisciplinary collaboration between cardiologists, hematologists, and imaging specialists are essential. Moreover, future research should focus on large-scale epidemiological studies to define the global burden of ATTR and AL cardiac amyloidosis and assess the efficacy of emerging treatments tailored to genetic variants. Table 4 shows the most recent articles about the diagnosis and management of cardiac amyloidosis.

In parallel with advances in diagnostic techniques, the therapeutic landscape for cardiac amyloidosis is evolving rapidly, driven by innovative approaches that go beyond traditional stabilizers. Transthyretin (TTR) silencers now include subcutaneous small interfering RNA (siRNA) agents, such as patisiran, which in the APOLLO-B trial demonstrated sustained reductions in NT-proBNP and hospitalization rates among ATTR cardiomyopathy patients [94], and vutrisiran, whose HELIOS-B study corroborated these benefits with an improved dosing profile and high tolerability [95]. Antisense oligonucleotides—exemplified by inotersen and its successor eplontersen (AKCEA-TTR-LRx)—have also shown a robust TTR knockdown, with eplontersen offering monthly dosing and a favorable safety signal [96]. On the stabilization front, acoramidis (AG10) received approval after phase 3 ATTRibute-CM results confirmed a marked symptomatic improvement and waveform normalization, complementing the longstanding tafamidis platform [97].

Perhaps the most transformative prospect lies in in vivo gene editing. Early first-in-human studies of NTLA-2001—a CRISPR/Cas9-based therapy—have achieved up to a 90% serum TTR reduction after a single infusion, underscoring the potential for a one-time curative approach [98]. Real-world anecdotes—such as the case of a septuagenarian treated by lipid–nanoparticle-delivered CRISPR machinery—highlight both the promise and delivery challenges of this modality. As these modalities mature, head-to-head and combination trials—especially those assessing long-term durability, off-target effects, and cardiac remodeling—will be crucial.

Collectively, these emerging therapies offer a spectrum of options—from lifelong stabilization and periodic silencing to potentially curative gene editing—that promise to transform cardiac amyloidosis from a relentlessly progressive disease into a manageable, and perhaps even reversible, condition. Continued multidisciplinary collaboration, patient access initiatives, and cost-effectiveness analyses will be essential to ensure the equitable delivery of these cutting-edge treatments.

To provide clinicians with an at-a-glance, evidence-based action plan for the subtype-specific management of cardiac amyloidosis, we have summarized the major society recommendations in Table 5. Disease-modifying therapies include transthyretin stabilizers (tafamidis; Class I, Level B) and RNA silencers (patisiran/inotersen; Class IIa, Level B) for ATTR amyloidosis [25,99], as well as proteasome-inhibitor-based chemotherapy (±daratumumab; Class I, Level B) and autologous stem-cell transplant (Class IIa, Level B) for AL amyloidosis [100,101]. Supportive care measures—diuretics (Class I, Level C), withholding β-blockers/ACE inhibitors in hypotensive restrictive physiology (Class IIb, Level C) and anticoagulation for atrial fibrillation or atrial dysfunction (Class I, Level C)—are drawn from the ESC heart failure and atrial fibrillation guidelines [73,102,103]. Finally, device and procedural interventions, such as TAVR for severe AS with concomitant CA (Class IIa, Level B) and ICD placement for the secondary prevention of VT/VF in AL amyloidosis (Class IIa, Level C), are recommended by the ACC/AHA and ACC expert consensus documents [99,104].

## 6. Limitations of the Study

Several limitations of this narrative review merit consideration. First, by design, our review was not conducted as a fully systematic or meta-analytic study; although we searched two major databases (PubMed and Scopus) over a defined interval, we did not apply formal risk-of-bias assessments or GRADE evidence grading, and we may have omitted relevant studies published outside the search window or in non-English journals. Second, the heterogeneity of the included studies—in terms of study populations, diagnostic criteria, imaging protocols, and therapeutic regimens—precludes a quantitative synthesis and limits the generalizability of our conclusions to specific patient subgroups or practice settings. Third, emerging therapies and diagnostic tools in cardiac amyloidosis are evolving rapidly; some of the most recent clinical trials and consensus statements may not yet be fully reflected, and cost-effectiveness considerations (particularly for high-cost agents such as tafamidis, inotersen, and diflunisal) were beyond the scope of our review. Finally, as with all narrative overviews, selection bias and publication bias—favoring positive or high-profile studies—may influence the balance of the evidence presented. Despite these limitations, our review aims to provide a comprehensive clinical perspective on current diagnostic advances and emerging therapies in cardiac amyloidosis, highlighting areas in need of further rigorous investigation.

## 7. Future Directions

Despite rapid advances in both diagnostic modalities and targeted treatments, several critical questions remain unanswered. First, robust, prospective risk-stratification models that integrate imaging metrics (ECV, strain patterns), biomarkers (NT-proBNP, troponin), genotypes (ATTRv vs. ATTRwt), and clinical features are lacking; developing and validating such multidimensional scores will be essential to identify patients most likely to benefit from early intervention. Second, with an expanding armamentarium—from TTR stabilizers (tafamidis and acoramidis) and RNA silencers (patisiran, vutrisiran, inotersen, and eplontersen) to in vivo gene editors—head-to-head or adaptive trials are urgently needed to define the optimal sequencing or combination of these agents, to clarify whether certain subgroups (for example, early-stage vs. advanced-stage ATTR-CA) derive greater incremental benefits from one class over another, and to assess long-term safety and durability. Third, real-world effectiveness and cost-effectiveness analyses—particularly in health systems with constrained resources—are scarce, yet crucial for informing policy, reimbursements, and equitable access. Finally, the field would benefit from large, multicenter registries and biobanks that can support translational research into novel biomarkers and mechanisms of disease progression. Addressing these gaps through collaborative, interdisciplinary efforts will be pivotal in transforming cardiac amyloidosis from an often-fatal disorder into a condition that can be reliably detected early, stratified by risk, and treated with precision.

## 8. Conclusions

Cardiac amyloidosis presents significant diagnostic and therapeutic challenges due to its heterogeneous presentations and underlying etiologies. Innovations in imaging modalities, molecular therapies, and interdisciplinary care models are reshaping the management paradigm and offering renewed hope for affected patients.

## Figures and Tables

**Table 1:** Clinical manifestations of AL and ATTR amyloidosis (in order of frequency).

Amyloidosis Subtype	Clinical Manifestations
**AL amyloidosis**	Renal dysfunction, proteinuria **Cardiomyopathy** Carpal tunnel Hepatic dysfunction Orthostatic hypotension Peripheral neuropathy Gastrointestinal abnormalities Hypothyroidism Macroglossia Periorbital purpura
**ATTR amyloidosis**	**Cardiomyopathy** Orthostatic hypotension Peripheral neuropathy Carpal tunnel Lumbar spinal stenosis Gastrointestinal abnormalities Spontaneous biceps tendon rupture Ocular floaters

**Table 2:** Main findings in cardiac amyloidosis.

Type of Assessment	Main Findings
**Clinical**	HF symptoms with prominent RV failure (peripheral congestion)
**Electrocardiography**	Low-voltage QRS (disproportional to LV wall thickness) Poor R-wave progression (pseudo-infarction pattern) Pathological Q-waves Atrial fibrillation AV conduction abnormalities Bundle branch block QT prolongation
**Echocardiography**	Unexplained LV hypertrophy Atrial dilatation RV free wall hypertrophy Myocardial granular sparkling (in non-harmonic imaging) or speckle appearance (in harmonic imaging) Thickening of AV valves and atrial septum Diastolic LV dysfunction Abnormal LV GLS with apical sparing (“cherry-on-top” pattern) Low-flow, low-gradient aortic stenosis
**Biomarkers**	Unexplained persistent low-level cTn elevation Significant BNP elevation (disproportional to HF symptoms)
**Cardiac magnetic resonance**	Diffuse subendocardial or transmural LGE High extracellular volume

**Table 3:** Scenarios that can guide the diagnostic pathway of cardiac amyloidosis.

Scenario	Findings	Interpretation	Next Steps
Scenario 1	Scintigraphy: no cardiac uptake. Monoclonal protein tests: negative.	Very low probability of cardiac amyloidosis; ATTR and AL amyloidosis are unlikely.	Consider alternative diagnoses. If suspicion persists, perform cardiac MRI (CMR) followed by cardiac or extracardiac biopsy, as scintigraphy may be negative in some ATTRv mutations (depending on TTR fibril composition) or rare subtypes of cardiac amyloidosis.
Scenario 2	Scintigraphy: cardiac uptake present. Monoclonal protein tests: negative.	Grade 2 or 3 cardiac uptake: ATTR cardiac amyloidosis confirmed; proceed with genetic testing to distinguish between ATTRv and ATTRwt. Grade 1 cardiac uptake: non-invasive diagnosis is not possible.	Histological confirmation of amyloid deposits (cardiac or extracardiac biopsy) is required.
Scenario 3	Scintigraphy: no cardiac uptake. Monoclonal protein tests: at least one abnormal.	AL amyloidosis must be ruled out.	Use CMR to confirm or exclude cardiac involvement. If CMR findings are supportive or inconclusive, perform cardiac or extracardiac biopsy for amyloid confirmation. If CMR findings do not support cardiac amyloidosis, diagnosis is unlikely. If CMR is unavailable, proceed directly to biopsy to avoid delays. Consultation with a hematologist is recommended.
Scenario 4	Scintigraphy: cardiac uptake present. Monoclonal protein tests: at least one abnormal.	Possible scenarios: ATTR with concomitant monoclonal gammopathy of undetermined significance (MGUS) or other hematological disorders producing FLC, AL amyloidosis, or coexistence of ATTR and AL amyloidosis.	Diagnosis requires histological confirmation and amyloid typing, typically through an endomyocardial biopsy.

**Table 4:** Systematic reviews and meta-analyses on the diagnosis of cardiac amyloidosis.

Title	Authors	Year	Study Type	Conclusion	Ref
Redefining the epidemiology of cardiac amyloidosis. A systematic review and meta-analysis of screening studies	Aimo et al.	2022	A systematic review and meta-analysis	Screening in certain clinical groups can identify a large number of patients who could benefit from treatment. ATTR-CA is the most common form of CA, but light-chain amyloidosis (AL-CA) is not rare, accounting for up to 18% of cases. The average age at diagnosis is between 74 and 90 years, and most patients are men (50–100%).	[66]
Cardiac amyloidosis imaging with amyloid positron emission tomography: A systematic review and meta-analysis	Kim et al.	2020	A systematic review and meta-analysis	Amyloid PET is a promising method for the diagnosis of cardiac amyloidosis, with high sensitivity and specificity. Semi-quantitative PET analysis helps differentiate between AL and ATTR, which is essential for selecting the appropriate treatment. The results support the use of PET in combination with other imaging techniques (MRI, bone scintigraphy) for the accurate diagnosis of CA.	[67]
Prevalence and clinical outcomes of ATTR: a systematic review and meta-analysis	Antonopoulos et al.	2022	A systematic review and meta-analysis	ATTR-CA is more common than previously thought, especially in populations with HFpEF, severe aortic stenosis, and hypertrophic cardiomyopathy. Survival depends on the ATTR subtype and genetic mutations. Patients with Val30Met have the worst prognosis. Tafamidis/patisiran therapy significantly improves survival, highlighting the importance of early diagnosis and appropriate treatment. Research gaps: additional epidemiological studies are needed to better understand the global distribution of ATTR.	[49]
Echocardiographic predictors of presence of cardiac amyloidosis in aortic stenosis	Jaiswal et al.	2022	A systematic review and meta-analysis	Echocardiography can help detect cardiac amyloidosis in patients with aortic stenosis, based on the parameters described above. Differences in wall thickness, LVMI, and diastolic parameters are useful for screening CA in patients with AS. Additional studies are needed to determine the optimal values of these parameters for more accurate differential diagnosis.	[51]
Concomitant transthyretin cardiac amyloidosis in patients undergoing TAVR for aortic stenosis: A systemic review and meta-analysis.	Fatima et al.	2024	A systematic review and meta-analysis	TTRCA is common in patients with severe aortic stenosis who require TAVR (13.3%). Patients with TTRCA have higher mortality and cardiovascular hospitalization rates compared to those without TTRCA, suggesting a more reserved prognosis. Larger studies are needed to determine the safety and efficacy of TAVR in patients with TTRCA, as current data come from small cohorts.	[63]
Prognostic Significance of Cardiac Amyloidosis in Patients With Aortic Stenosis: A Systematic Review and Meta-Analysis	Ricci et al.	2021	A systematic review and meta-analysis	In summary, transthyretin cardiac amyloidosis (CA) is associated with a significantly increased risk of all-cause mortality in elderly patients with aortic stenosis (AS). Additionally, maximum left ventricular wall thickness (LVWT) appears to be a key prognostic factor in individuals with both conditions, independent of age, left ventricular ejection fraction, and aortic valve replacement. Until further evidence from randomized controlled trials clarifies the effectiveness of amyloid-targeted and valve-focused therapies across varying disease severity phenotypes, treatment decisions for patients with dual pathology should be carefully assessed by the local heart team, with a personalized discussion of the benefit–risk balance for each patient.	[68]
Tissue mapping by cardiac magnetic resonance imaging for the prognostication of cardiac amyloidosis: A systematic review and meta-analysis	Cai et al.	2024	A systematic review and meta-analysis	The study concludes that cardiac magnetic resonance (CMR) tissue mapping is a valuable prognostic tool for assessing disease severity and predicting mortality in patients with cardiac amyloidosis. Higher native T1 times, increased extracellular volume (ECV), and lower myocardial-to-skeletal T2 ratios are significantly associated with worse outcomes. These findings support the clinical utility of CMR-based tissue characterization in risk stratification and guiding treatment decisions for patients with cardiac amyloidosis.	[69]
Prognostic impact of light-chain and transthyretin-related categories in cardiac amyloidosis: A systematic review and meta-analysis	Xin et al.	2019	A systematic review and meta-analysis	The study underscores the importance of distinguishing between amyloidosis subtypes, as they have distinct prognostic implications.	[58]
Prevalence and Risk Factors of Cardiac Amyloidosis in Heart Failure: A Systematic Review and Meta-Analysis	See et al.	2022	A systematic review and meta-analysis	The findings underscore the importance of maintaining a high index of suspicion for CA in HF patients, especially those presenting with the identified risk factors. Early recognition and appropriate diagnostic evaluations are crucial, as timely diagnosis of CA can significantly influence management strategies and improve patient outcomes.	[70]
Prevalence and outcomes of concomitant cardiac amyloidosis and aortic stenosis: A systematic review and meta-analysis	Ho et al.	2021	A systematic review and meta-analysis	The findings underscore the importance of considering the coexistence of CA in patients with AS, as this dual pathology is linked to poorer prognoses. Early detection and tailored management strategies are crucial to improve outcomes in this patient population.	[71]
Diagnostic accuracy of bone scintigraphy imaging for transthyretin cardiac amyloidosis: systematic review and meta-analysis	Ahluwalia et al.	2023	A systematic review and meta-analysis	Bone scintigraphy imaging is highly accurate for identifying patients with ATTR-CM. The slight differences in specificity among the diagnostic approaches may have clinical implications, especially when applied to low-risk screening populations. The study highlights the importance of considering disease prevalence when interpreting diagnostic accuracy and suggests that quantitative analysis of SPECT imaging may offer the highest specificity among the evaluated methods.	[72]
Diagnostic performance of imaging investigations in detecting and differentiating cardiac amyloidosis: a systematic review and meta-analysis	Brownrigg et al.	2019	A systematic review and meta-analysis	The study concludes that while both CMR and nuclear scintigraphy are valuable tools for detecting cardiac amyloidosis, nuclear scintigraphy, especially when paired with monoclonal protein screening, offers superior performance in differentiating ATTR from AL amyloidosis. These findings support the integration of both imaging modalities into a non-invasive diagnostic algorithm for CA.	[32]
Tolerability and effectiveness of beta-blockers in patients with cardiac amyloidosis: A systematic review and meta-analysis	Kang et al.	2024	A systematic review and meta-analysis	The findings suggest that BB therapy may have limited effectiveness and tolerability in patients with cardiac amyloidosis. Clinicians are advised to exercise caution when prescribing BBs to this patient population, considering potential adverse effects and closely monitoring for signs of intolerance. Alternative therapeutic strategies should be explored to manage heart failure symptoms in CA patients. These conclusions align with previous research indicating challenges in BB tolerability among CA patients. For instance, a study published in *Frontiers in Cardiovascular Medicine* reported that over half of the patients prescribed BBs discontinued therapy due to adverse effects such as hypotension and bradycardia.	[73]
Aortic valve intervention for aortic stenosis and cardiac amyloidosis: a systematic review and meta-analysis	de Campos et al.	2022	A systematic review and meta-analysis	The findings suggest that aortic valve interventions can be beneficial for patients with both AS and CA, offering symptomatic improvement and potential survival benefits. However, the associated mortality rates underscore the importance of careful patient selection and comprehensive preoperative evaluation. Decisions regarding such interventions should involve a multidisciplinary heart team approach, considering the individual patient’s clinical condition and comorbidities. These conclusions align with previous research indicating that while aortic valve interventions in patients with AS and CA carry certain risks, they can lead to meaningful improvements in patient outcomes when appropriately applied.	[74]
Effect of beta-blockade on mortality in patients with cardiac amyloidosis: A systematic review and meta-analysis	Kwok et al.	2024	A systematic review and meta-analysis	The study concludes that beta-blocker therapy does not confer a mortality benefit in patients with cardiac amyloidosis and may be associated with potential harm, particularly in those with AL amyloidosis. These findings suggest that the routine use of beta-blockers in CA patients should be carefully reconsidered, and alternative therapeutic strategies may be warranted. Clinicians are advised to exercise caution when prescribing beta-blockers to this patient population and to monitor for adverse effects diligently.	[75]
Artificial intelligence-enhanced electrocardiogram for the diagnosis of cardiac amyloidosis: A systemic review and meta-analysis.	Khan et al.	2024	A systematic review and meta-analysis	The findings suggest that AI-enhanced ECG models are effective tools for detecting CA and its subtypes. These models may facilitate early diagnosis and intervention, potentially improving patient outcomes. The study highlights the promise of integrating AI with ECG analysis to enhance the non-invasive detection of cardiac amyloidosis.	[76]
Transcatheter aortic valve replacement in aortic stenosis and cardiac amyloidosis: a systematic review and meta-analysis	Cannata et al.	2022	A systematic review and meta-analysis	The study concludes that TAVR is an effective and safe treatment option for patients with concomitant AS and CA, offering a significant survival advantage over medical therapy alone. The safety profile of TAVR in these patients is comparable to those with AS alone, except for a non-significant trend toward a higher risk of permanent pacemaker implantation. These findings support the consideration of TAVR as a viable therapeutic option in this patient population.	[77]
The value of the electrocardiogram in the recognition of cardiac amyloidosis: a systematic meta-analysis.	Sun et al.	2024	A systematic review and meta-analysis	The study concludes that while ECGs exhibit high specificity in detecting cardiac amyloidosis, their sensitivity is relatively low. This implies that a positive ECG finding is quite reliable for diagnosing CA; however, a negative ECG does not definitively rule out the disease. Therefore, ECGs can be a valuable initial screening tool, but they should be complemented with other diagnostic methods, such as echocardiography, cardiac magnetic resonance imaging, or biopsy, to ensure accurate detection and diagnosis of cardiac amyloidosis.	[78]
Effect of ICD implantation on cardiovascular outcomes in patients with cardiac amyloidosis: A systematic review and meta-anaylsis	Halawa et al.	2020	A systematic review and meta-analysis	The study suggests that while ICDs can effectively manage ventricular arrhythmias in CA patients, the overall mortality remains high, and the benefit of ICD implantation may be limited to specific subgroups. These findings underscore the importance of individualized patient assessment when considering ICD therapy in the context of cardiac amyloidosis. These conclusions align with previous research indicating that ICDs may not significantly improve survival in CA patients, despite their efficacy in terminating arrhythmias. For instance, a study published in *Europace* in 2020 reported that ICD implantation in CA patients was not associated with longer survival, despite comparable event rates to non-CA patients. Given the complex nature of cardiac amyloidosis and the limited survival benefit observed with ICD therapy, a comprehensive, multidisciplinary approach is essential to optimize patient outcomes.	[79]
Monitoring the Efficacy of Tafamidis in ATTR Cardiac Amyloidosis by MRI-ECV: A Systematic Review and Meta-Analysis	Kato et al.	2024	A systematic review and meta-analysis	The findings suggest that MRI-ECV is a valuable non-invasive imaging biomarker for monitoring the efficacy of tafamidis in patients with ATTR-CM. The stability of MRI-ECV in tafamidis-treated patients indicates that the therapy effectively halts disease progression, whereas increases in MRI-ECV in untreated patients reflect ongoing disease advancement. This underscores the potential of MRI-ECV measurements in guiding clinical decisions and assessing therapeutic responses in ATTR-CM management.	[80]
Diagnostic performance of PET for detection of cardiac amyloidosis: A systematic review and meta-analysis	Kim et al.	2020	A systematic review and meta-analysis	The findings suggest that PET imaging is a highly sensitive and specific modality for the detection of cardiac amyloidosis. The significant difference in tracer uptake between CA patients and controls underscores the potential of PET as a valuable tool in the non-invasive diagnosis of this condition. These results support the integration of PET into clinical practice for the evaluation of suspected cardiac amyloidosis.	[53]
Diagnostic Sensitivity of Abdominal Fat Aspiration Biopsy for Cardiac Amyloidosis: A Systematic Review and Meta-Analysis	Wang et al.	2024	A systematic review and meta-analysis	The findings suggest that while abdominal fat aspiration biopsy is a valuable diagnostic tool for light-chain amyloidosis cardiomyopathy, its effectiveness is limited in diagnosing ATTR cardiomyopathy. Therefore, clinicians should consider alternative or additional diagnostic approaches when ATTR-CM is suspected.	[81]
Role of biomarkers in early diagnosis and prognosis of cardiac amyloidosis: A systematic review and meta-analysis	Albulushi et al.	2025	A systematic review and meta-analysis	The findings underscore the critical role of biomarkers in the early diagnosis and prognosis of cardiac amyloidosis. NT-proBNP and troponins are well-established markers for early detection, while novel biomarkers offer additional insights into disease progression and subtype differentiation. Integrating biomarker analysis with imaging studies may enhance diagnostic accuracy and inform treatment strategies for CA patients.	[82]
Native T1 Mapping, Extracellular Volume Mapping, and Late Gadolinium Enhancement in Cardiac Amyloidosis: A Meta-Analysis	Pan et al.	2020	A systematic review and meta-analysis	The findings suggest that ECV mapping offers superior diagnostic and prognostic utility in the assessment of cardiac amyloidosis compared to LGE and native T1 mapping. While native T1 mapping provides similar sensitivity and specificity to LGE without the need for contrast agents, ECV mapping stands out as the most effective technique for both diagnosing CA and predicting adverse outcomes. These results support the integration of ECV mapping into clinical practice for comprehensive evaluation of patients with suspected or confirmed cardiac amyloidosis.	[19]
Diagnostic accuracy of bone scintigraphy in the assessment of cardiac transthyretin-related amyloidosis: a bivariate meta-analysis	Treglia et al.	2018	A systematic review and meta-analysis	The findings suggest that bone scintigraphy with technetium-labeled radiotracers is a highly accurate non-invasive method for diagnosing cardiac transthyretin amyloidosis. The high sensitivity and specificity support its use as a reliable diagnostic tool in clinical practice.	[83]
Diagnostic performance of CMR, SPECT, and PET imaging for the detection of cardiac amyloidosis: a meta-analysis.	Wu and Yu	2021	A systematic review and meta-analysis	The findings suggest that SPECT imaging exhibits superior diagnostic performance in detecting cardiac amyloidosis compared to CMR and PET. However, all three imaging techniques are valuable tools in the non-invasive diagnosis of CA. The study highlights the importance of selecting the appropriate imaging modality based on clinical context, availability, and patient-specific factors.	[84]
An analysis regarding the article “Artificial intelligence-enhanced electrocardiogram for the diagnosis of cardiac amyloidosis: A systemic review and meta-analysis	Su and Leng	2024	A systematic review and meta-analysis	This analysis contributes to the ongoing discourse on the role of AI in cardiology by highlighting the strengths and limitations of current research on AI-enhanced ECGs for CA diagnosis. It underscores the importance of rigorous methodological standards and the need for continued investigation to ensure the safe and effective integration of AI tools into clinical practice.	[85]
Performance of bone tracer for diagnosis and differentiation of transthyretin cardiac amyloidosis: a systematic review and meta-analysis	Zhao et al.	2021	A systematic review and meta-analysis	The findings suggest that bone scintigraphy is a valuable non-invasive tool with high accuracy for diagnosing transthyretin cardiac amyloidosis. While it plays a modest role in differentiating ATTR-CA from AL-CA, certain tracers like ^18^F-NaF may offer enhanced differentiation capabilities. These results support the use of bone scintigraphy in clinical practice for the assessment of suspected cardiac amyloidosis.	[86]
Transcatheter Aortic Valve Implantation in Cardiac Amyloidosis and Aortic Stenosis	Riley et al.	2023	A systematic review and meta-analysis	The findings suggest that TAVI may offer a survival advantage over conservative medical therapy in patients with concomitant AS and CA. This supports the consideration of TAVI as a viable treatment option in this patient population. However, the authors note that the evidence is derived from a limited number of observational studies, underscoring the need for further research to confirm these results and to establish comprehensive management guidelines for patients with both AS and CA.	[87]
Prognostic Value of Bone Scintigraphy in Cardiac Amyloidosis: A Systematic Review and Meta-analysis	Cho and Han	2025	A systematic review and meta-analysis	The findings suggest that bone scintigraphy not only serves as a diagnostic tool for cardiac amyloidosis but also provides valuable prognostic information. Specific imaging biomarkers derived from bone scintigraphy can stratify patients based on risk, thereby guiding clinical decision-making and management strategies. The study underscores the importance of incorporating bone scintigraphy into the routine evaluation of patients with suspected or confirmed cardiac amyloidosis to inform prognosis and tailor therapeutic approaches.	[88]
Diagnostic Role of NT-proBNP in Patients with Cardiac Amyloidosis Involvement: A Meta-Analysis	Zhang and Chaolu	2022	A systematic review and meta-analysis	The findings suggest that NT-proBNP is a valuable biomarker for the early diagnosis of cardiac involvement in amyloidosis patients, demonstrating high sensitivity and specificity. Its use can aid clinicians in identifying cardiac amyloidosis, potentially leading to earlier interventions and improved patient outcomes.	[89]
Diagnostic accuracy of cardiovascular magnetic resonance for patients with suspected cardiac amyloidosis: a systematic review and meta-analysis.	Zhao et al.	2016	A systematic review and meta-analysis	The findings suggest that CMR is a valuable non-invasive tool for diagnosing cardiac amyloidosis, offering high sensitivity and specificity. The use of advanced CMR techniques, such as LGE and T1 mapping, enhances the detection of myocardial amyloid infiltration, thereby aiding in the early diagnosis and management of CA.	[90]
Meta-analysis of post-transcatheter aortic valve replacement outcomes in patients with cardiac amyloidosis and aortic stenosis	Jaiswal et al.	2023	A systematic review and meta-analysis	The findings suggest that patients with concomitant aortic stenosis and cardiac amyloidosis undergoing TAVR may face higher risks of adverse outcomes, including increased mortality within 30 days, stroke, acute kidney injury, and major bleeding events, compared to patients with aortic stenosis alone. These results underscore the importance of careful patient selection and risk stratification when considering TAVR for individuals with both AS and CA. Further research is warranted to develop strategies to mitigate these risks and improve outcomes in this patient population.	[91]
Diagnostic Accuracy and Prognostic Value of Relative Apical Sparing in Cardiac Amyloidosis—Systematic Review and Meta-Analysis	Lee et al.	2024	A systematic review and meta-analysis	The findings suggest that the relative apical sparing pattern in echocardiographic strain imaging is a valuable tool for both the diagnosis and prognosis of cardiac amyloidosis. Its application in clinical practice can aid in the early detection and management of CA, potentially leading to improved patient outcomes.	[92]
Diagnostic and Prognostic Utility of Native T1 Mapping and Extracellular Volume for Cardiac Amyloidosis: A Meta-Analysis	Wang et al.	2021	A systematic review and meta-analysis	The findings suggest that native T1 mapping and ECV are valuable tools in the diagnosis and prognosis of cardiac amyloidosis. Establishing specific reference ranges enhances their clinical applicability, aiding in early detection and risk stratification of patients with CA.	[93]

**Table 5:** Guideline-based management recommendations.

Intervention	Indication	Class/Level	Refference
**Tafamidis**	Symptomatic ATTR-CA (NYHA I–II)	I B	[25,104]
**Patisiran/Inotersen**	ATTRv with polyneuropathy (±cardiac involvement)	IIa B	[99]
**Diflunisal**	ATTR-CA when tafamidis unavailable or intolerant	IIb C	[25]
**Bortezomib-based chemo (±daratumumab)**	AL-CA with hematologic involvement	I B	[100]
**Autologous stem-cell transplant**	Selected low-risk AL-CA patients	IIa B	[101]
**Diuretics**	Volume overload	I C	[102]
**Avoid β-blockers/ACE-I**	Hypotension in restrictive physiology	IIb C	[73]
**Anticoagulation**	AF or atrial dysfunction in CA	I C	[103]
**TAVR**	Severe AS + CA (NYHA II–III)	IIa B	[105]
**ICD**	Secondary prevention in AL-CA with sustained VT/VF	IIa C	[99]

ATTR-CA, transthyretin cardiac amyloidosis; AL-CA, immunoglobulin light-chain cardiac amyloidosis; NYHA, New York Heart Association; VT, ventricular tachycardia; VF, ventricular fibrillation; TAVR, transcatheter aortic valve replacement; ACE-I, angiotensin-converting enzyme inhibitor; ICD, implantable cardioverter–defibrillator; and Class/Level: guideline recommendation class and level of evidence.
